# The complete mitochondrial genome of *Syrphus ribesii* (Diptera: Syrphoidea: Syrphidae)

**DOI:** 10.1080/23802359.2021.1872446

**Published:** 2021-02-11

**Authors:** Mengchen Chen, Ke Peng, Chengyong Su, Yunliang Wang, Jiasheng Hao

**Affiliations:** Laboratory of Molecular Evolution and Biodiversity, College of Life Sciences, Anhui Normal University, Anhui, Wuhu, P. R. China

**Keywords:** Mitochondrial genome, phylogeny, Cyclorrhapha, Syrphoidea, *Syrphus ribesii*

## Abstract

The complete mitochondrial genome of *Syrphus ribesii* was determined in this study. The double-stranded circular DNA molecule was 16,530 bp in length, containing 37 typical genes: 13 protein-coding genes (PCGs), 2 rRNA genes, 22 tRNA genes, and an A + T-rich region. Thirteen PCGs were 11,196 bp in size, encoding 3720 amino acids in total. All the PCGs started with ATN, except the *COI* used TTG as its initiation codon. Most PCGs terminated with standard codon TAA, while the *COI* ended with T and the *ND5* ended with TA. The *lrRNA* and *srRNA* genes were 1341 bp and 793 bp in length, respectively. The A + T-rich region harbored some typical structures characteristic of the dipterans. The phylogenetic tree showed that *Syrphus ribesii* was closely related to *Eupeodes corollae*, and the Syrphidae and Pipunculidae constituted a monophyletic group within the Syrphoidea.

As one of the most abundant groups in Diptera, Syrphidae (Cyclorrhapha) is traditionally divided into three subfamilies (Eristalinae, Microdontinae, Syrphinae) covering about 230 genera, 6000 living species distributed nearly all around the world (Sommaggio 1999; Nedeljković et al. 2013). Most adults of Syrphidae have the habit of visiting flowers, making them one of the most important pollinators (Reemer 2013; Li 2019). However, to date, only about 20 complete mitochondrial genomes of Syrphidae have been determined (Pu et al. 2017; Li and Li 2020).

In this study, we determined the complete mitochondrial genome of *Syrphus ribesii* (Linnaeus, 1758), and conducted the phylogenetic analysis of the Syrphidae with other related groups. Adult individuals of *Syrphus ribesii* were collected from Shannan, Tibet Autonomous Region, China (91°76′E, 29°23'N) in July 2017. After sample collection, the fresh tissues were preserved in absolute ethanol immediately for fixation and stored at −20 °C in the Entomological Specimen Room of Anhui Normal University (Jiasheng Hao, jshaonigpas@sina.com) under the voucher number ANUN-201707-1. The whole genomic DNA was extracted from thoracic muscle using the Animal Genome DNA Extraction Kit (Shanghai Sangon Biotech Co., Ltd., China), following the manufacturer’s protocols. The mitogenome of *Syrphus ribesii* was generated by amplified overlapping fragments using 3 pairs of universal PCR primers (Park et al. 2012) and a set of newly designed primers. PCR primers are available upon request. All fragments were sequenced on an ABI 3730XL DNA sequencer by Sangon Biotechnology Company (Shanghai, China) with primers walking on both strands. Sequences obtained were proofread and assembled using BioEdit v7.0.5 (Hall et al. 2011) and SeqMan program was included in the Lasergene software package (DNAStar, Inc., USA, NewYork). PCGs, rRNAs and the A + T-rich region were confirmed by the boundaries of tRNAs, and by alignment with other Syrphidae gene sequences using MEGA 7.0 (Kumar et al. 2016). The tRNA genes were identified by the MITOS2 WebServer (Bernt et al. 2013).

The complete mitogenome sequence of *Syrphus ribesii* was 16,530 bp in length (GenBank accession No. MW091497), including 13 protein-coding genes (PCGs), 22 transfer RNA (tRNA) genes, 2 ribosomal RNA (rRNA) genes, and an A + T-rich region. The gene arrangement and orientation were identical to other congeneric insects (Li et al. 2017; Chen et al. 2020). The base composition of the whole genome was detected to be 40.7% A, 40.4% T, 8.3% G and 10.7% C, exhibiting a relatively strong AT bias. Thirteen PCGs were 11,196 bp in size, encoding 3720 amino acids in total. All 13 PCGs started with the typical ATN codons, except for *COI* which was initiated by TTG. Eleven PCGs used universal TAA as their termination codons, whereas *COI* and *ND5* used single T and TA as their stop codons, respectively. All tRNAs were well folded into a clover-leaf secondary structure except for *tRNA^Ser^* (AGN) losing the dihydrouridine (DHU) arm. The *lrRNA* and *srRNA* genes were 1341 bp and 793 bp in length, respectively. The A + T-rich region was 1491 bp in size, harboring some typical structures characteristic of dipterans: a 20 bp of poly-T stretch approximately in the center of the A + T-rich region and a microsatellite-like repeat (TA)_9_ located 127 bp upstream of the *srRNA* (Li et al. 2015; Zhang et al. 2016).

The phylogenetic analysis of the 45 Cyclorrhapha species was conducted based on the concatenated 13 PCG nucleotide sequence data with the maximum likelihood (ML) method, using two species from Tabanidae and Nemestrinidae as the outgroups (Figure 1). The ML tree was inferred by IQ-TREE v1.6.8 (Nguyen et al. 2015) using the best-fitting GTR + I + G model which was estimated with ModelFinder (Kalyaanamoorthy et al. 2017) following the BIC criterion. The bootstrap support (BS) values of the tree nodes were calculated using 1000 replicates. The result indicated that *Syrphus ribesii* was closely related to *Eupeodes corollae* within the family Syrphidae. The Syrphidae and Pipunculidae formed a monophyletic group within the Syrphoidea, whereas the two families were shown to be paraphyletic in the previous study based on larger-scale transcriptomic data (Pauli et al. 2018).

**Figure 1.**
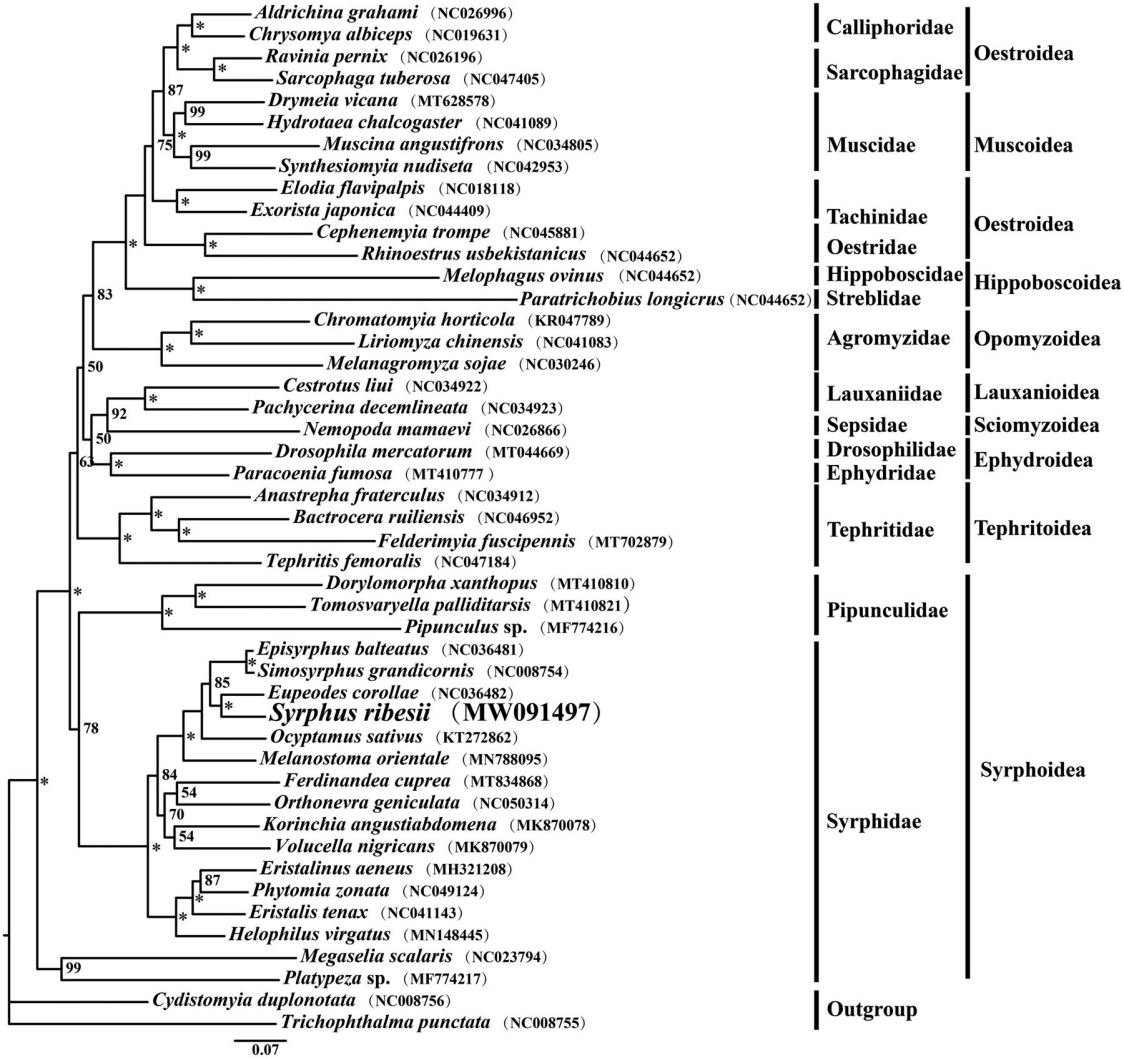
The maximum-likelihood (ML) phylogenetic tree of 45 Cyclorrhapha species inferred from the concatenated 13 PCG nucleotide sequence data. The numbers on each tree node represents the bootstrap values (*BS = 100%). The alphanumeric characters in parentheses indicate the GenBank accession numbers.

## Data Availability

The data that support the findings of this study are openly available in NCBI (National Center for Biotechnology Information) at https://www.ncbi.nlm.nih.gov/, reference number MW091497.
